# The extensive networks of frequent population mobility in the Samoan Islands and their implications for infectious disease transmission

**DOI:** 10.1038/s41598-018-28081-x

**Published:** 2018-06-28

**Authors:** Zhijing Xu, Colleen L. Lau, Xiaoyan Zhou, Saipale Fuimaono, Ricardo J. Soares Magalhães, Patricia M. Graves

**Affiliations:** 1 Research School of Population Health, The Australian National University, ACT 2601 Canberra, Australia; 2 Children’s Health and Environment Program, Child Health Research Centre, The University of Queensland, South Brisbane, 4101 QLD Australia; 3 UQ Spatial Epidemiology Laboratory, School of Veterinary Science, The University of Queensland, Gatton, 4343 QLD Australia; 4 Department of Health, Pago Pago, American Samoa USA; 5 Australian Institute of Tropical Health and Medicine, College of Public Health, Medical and Veterinary Sciences, James Cook University, Cairns, Queensland Australia

## Abstract

Population mobility has been demonstrated to contribute to the persistent transmission and global diffusion of epidemics. In the Pacific Islands, population mobility is particularly important for emerging infectious diseases, disease elimination programs, and diseases spread by close contact. The extent of population mobility between American Samoa villages, Samoa districts and other countries was investigated based on travel data collected during community surveys in American Samoa in 2010 and 2014. Within American Samoa, workers commuted daily across the whole of the main island of Tutuila, with work hubs drawing from villages across the island. Of the 670 adult workers surveyed, 37% had traveled overseas in the past year, with 68% of trips to Samoa. Of children aged 8–13 years (*n* = 337), 57% had traveled overseas, with 55% of trips to Samoa. An extensive network of connections between American Samoa villages and Samoa districts was demonstrated, with most trips lasting one week to one month. Our study showed that populations in the Samoan islands are highly mobile, and quantified the extent and destinations of their travels. Our findings offer insight into the impact of population mobility on the transmission of infectious diseases and data to refine existing models of disease transmission in the Pacific islands.

## Introduction

Population mobility has long been a focus of research and programmatic activities related to infectious disease prevention and control, given its crucial role in disease propagation, emergence and outbreaks^1–3^. Historically, population mobility has contributed to the persistent transmission of malaria, and eradication cannot be achieved without considering this factor^4^. More recently, the aviation network has been demonstrated to facilitate the global spread of infectious diseases, such as pandemic influenza^5,6^. Furthermore, seasonal population movements are believed to be able to cause systematic biases in surveillance estimates of disease burden, leading to misallocation of resources^7^. The analysis of the drivers of infectious disease threat events (IDTEs) collected at the European Center for Disease Prevention and Control between 2008 and 2013 indicated that travel and tourism ranked above all other driver categories in contributing to the IDTEs^8^. Balcan *et al*. further showed how the interplay between short-scale commuting flows and long-range airline traffic together shaped the spatiotemporal pattern of global epidemics^9^.

Infectious diseases are responsible for significant morbidity and mortality in the Pacific Islands, including the Samoan Islands^10–13^. Travel between the islands is likely to be a major driver of the spread of IDTEs in the region, but there are currently few empirical data available on travel and migration patterns, or assessment of use of such data to inform models of the spread of infectious diseases in the region. In the Pacific Islands, population mobility is particularly important for emerging infectious diseases, disease elimination programs, and diseases spread by close personal contact. Firstly, large outbreaks of emerging vector-borne infectious diseases, e.g. Zika, chikungunya, and dengue, have spread across the Pacific Islands in recent years^14–18^ and population mobility is believed to have contributed to the increase in diversity, frequency, spread, and geographic extent of the recent arbovirus outbreaks^12^. Secondly, certain diseases are targeted for elimination in the region, such as lymphatic filariasis^11^, trachoma^19^, and malaria^20^ (in Melanesian countries). Infected travellers can reintroduce pathogens to areas that have reduced disease prevalence to low levels, and threaten the success of elimination programs. For lymphatic filariasis, research evidence from American Samoa suggests that transmission is still occurring and has likely given rise to new infections^21–23^ even though transmission assessment surveys have been passed in 2011 and 2015^24^. Residual geographical “hotspots” of increased prevalence may be important for maintaining transmission^23^. Studies of population movement may shed light on one of the reasons for persistent transmission, and whether the intensity of travel between the islands might be a significant source of parasite reintroduction. Lastly, population mobility is important for diseases transmitted through close personal contact, e.g. the spread of measles and influenza via respiratory droplets^25^. Although models have been developed to inform the size and characteristics of transmission networks and potential control measures^26,27^, the lack of high-resolution population mobility data has limited their practical applications as they are unable to incorporate the influence of short-scale commuting flux.

The study aims to use survey data to investigate the population mobility between locations (villages or districts) in American Samoa (a US Territory in the South Pacific) and its most connected neighbouring countries, particularly Samoa (an independent country). Understanding of population mobility will be crucial for informing models of infectious disease transmission in the Samoan islands, including refinements of existing models of infectious disease transmission in Pacific islands^26–29^.

## Results

### Mobility within American Samoa

#### Network A - daily commuting of adults in 2010

**Figure 1:**
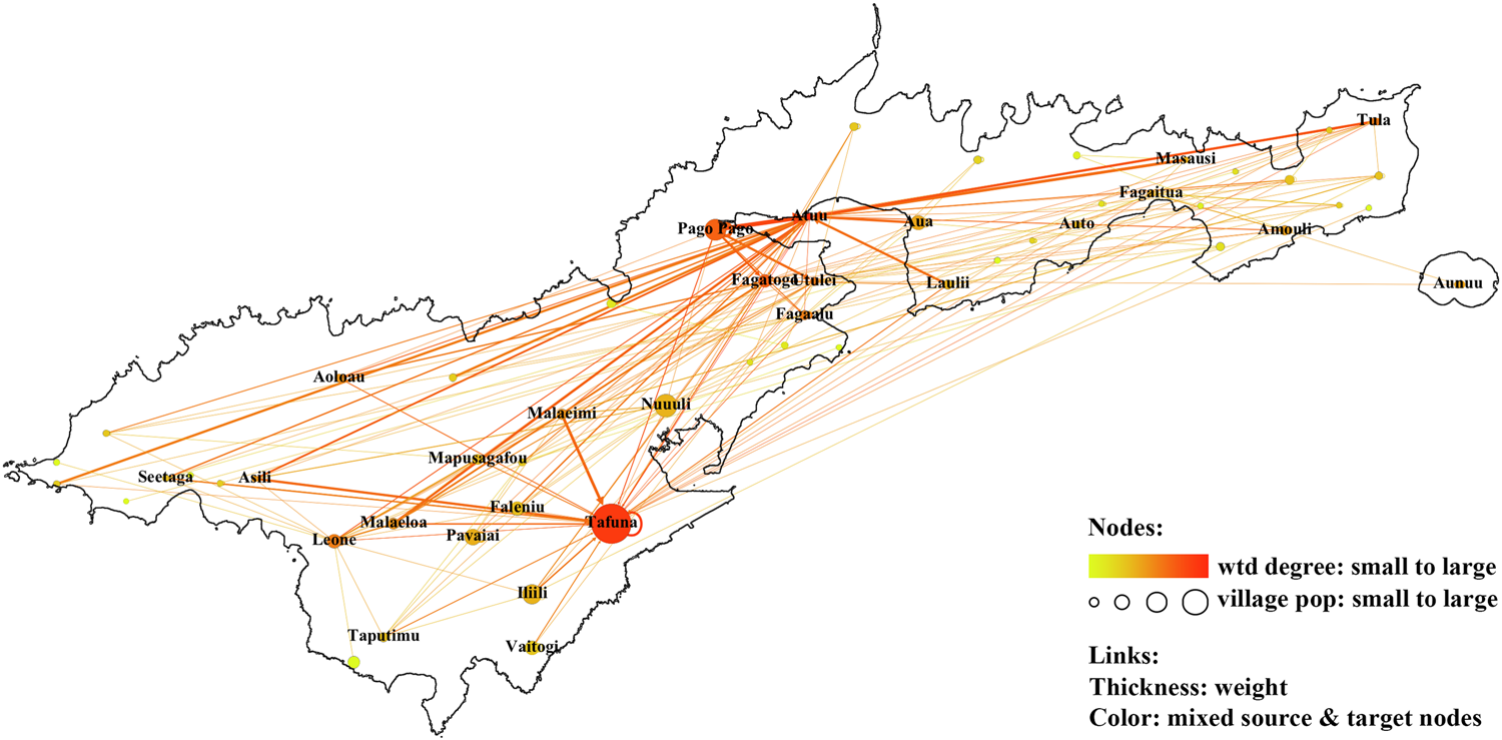
Network A: Commuting between residence and workplace villages within American Samoa, 2010. The color of lines is an average of weight of source and target nodes. wtd degree: weighted degree.

In 2010, 423 of 807 respondents in a community survey reported being employed, and 411 of them provided information on the workplace village. The directed commuting network from the residence villages to the workplace villages was produced (Fig. 1). The population covered by the network was 51,864, about 93.4% of the total population in American Samoa. The degree centrality was investigated in Supplementary Fig. S1. Both the in-degree and the weighted in-degree were highly skewed, indicating that a small number of villages attracted the majority of commuters. The commuting hubs were villages with the largest populations, such as Tafuna, Pago Pago and Fagatogo, plus the village of Atu’u. Despite having a very small population (only 359 in the 2010 population census), Atu’u was found to be the most important commuting hub with the largest weighted degree in the network because the tuna cannery located there was one of the largest employers in American Samoa.

The 2010 survey indicated an employment ratio (the proportion of adults currently in paid employment) of 52.4% (423/807) in 2010, but this is likely to be an underestimate because the survey was undertaken in communities during daytime. The employment ratio of males (58.6%, 248/423) was significantly higher than that of females (45.8%, 174/380) (*RR* = 1.28 and *p* = 0.013).

The age distribution of workers in the 2010 survey and the employment ratio by age group were analyzed in Supplementary Fig. S2 and Supplementary Table S1. People aged between 15–24 years and ≥65 years were found to work significantly less than other age groups. In American Samoa, many 15–18 year-olds would still be in full-time education.

**Figure 2:**
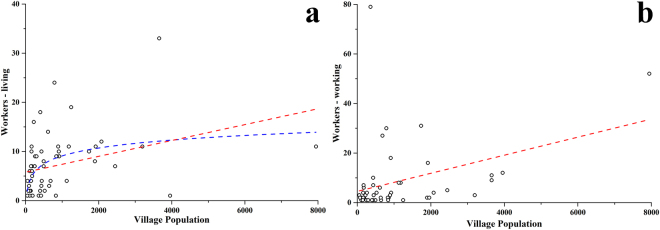
Correlation between the number of workers living or working in particular villages, and village population, 2010. Each point represents a village. (**a**) the number of workers living in the village, (i) red dashed line: *y* = 5.781 + 0.00161*x* and (ii) blue dashed line: *y* = −6.927 − 2.320 ln (*x* − 1.963); (**b**) the number of workers working in the village, red dashed line: *y* = 4.541 + 0.00365*x*.

The correlation between the number of workers living in particular villages and the village population was analyzed to investigate the commuting patterns in American Samoa. A significant positive correlation was found between the number of workers living in a village and the village size (Fig. 2(a)) and between the number of workers working in a village and the village size (Fig. 2(b)). However, there was no good fit with either linear (red dashed line) or nonlinear (blue dashed line) functions indicating that either sample size per village was insufficient for detecting significant patterns, or that there may be other factors (such as road distance) influencing the connections between work and residence villages.

#### Daily commuting of cannery workers in 2014

The largest non-government employer in American Samoa, with approximately 2,500 employees in 2014, was the Starkist tuna cannery. In 2014, we interviewed 498 workers at the tuna cannery to elaborate on the commuting network developed in 2010 from household interviews, and to understand the demographic characteristics and movements of this important segment of the population. These workers were interviewed at their work site, so the commuting network can only be developed in one direction (from home to one work site). The cannery workers resided in 48 villages and reported a total of 541 jobs in 14 villages (some respondents had more than one job in the past 12 months). Supplementary Fig. S3 shows the commuting network of the workers in the 2014 survey.

Besides two unspecified-gender respondents, 37.1% (184) of cannery workers surveyed were males and 62.9% (312) were females. In the 2010 census, the total numbers of males and females aged ≥15 years were 18,069 and 18,025 respectively. The Poisson test indicated there was a higher proportion of females than males in the surveyed cannery workers (*RR* = 0.59, *p* < 0.001). The cannery workers were grouped by their residence villages (Supplementary Fig. S4). The age structure of the cannery workers sampled was analyzed and compared to the general age structure of the 2010 census population (Supplementary Fig. S5(a)). The largest age group in the cannery workers was people aged 35–54 years (64.3%), followed by those aged 15–24 years. A positive correlation was found between the number of cannery workers from a particular village and the village population based on the regression analysis in Supplementary Fig. S5(b).

#### Residence Relocation

Besides the daily commuting between home and work, the permanent residential relocations between villages in American Samoa in the last year were also investigated. Of 670 adult workers, 18 (2.7%) individuals reported a total of 21 relocations, of which 66.7% were to larger villages. Pago Pago was the most important hub, with eight individuals moving in or out (Supplementary Fig. S6). Thus, frequency of relocation between villages in American Samoa was low.

### Network B – Travelling Overseas

#### Children

**Figure 3:**
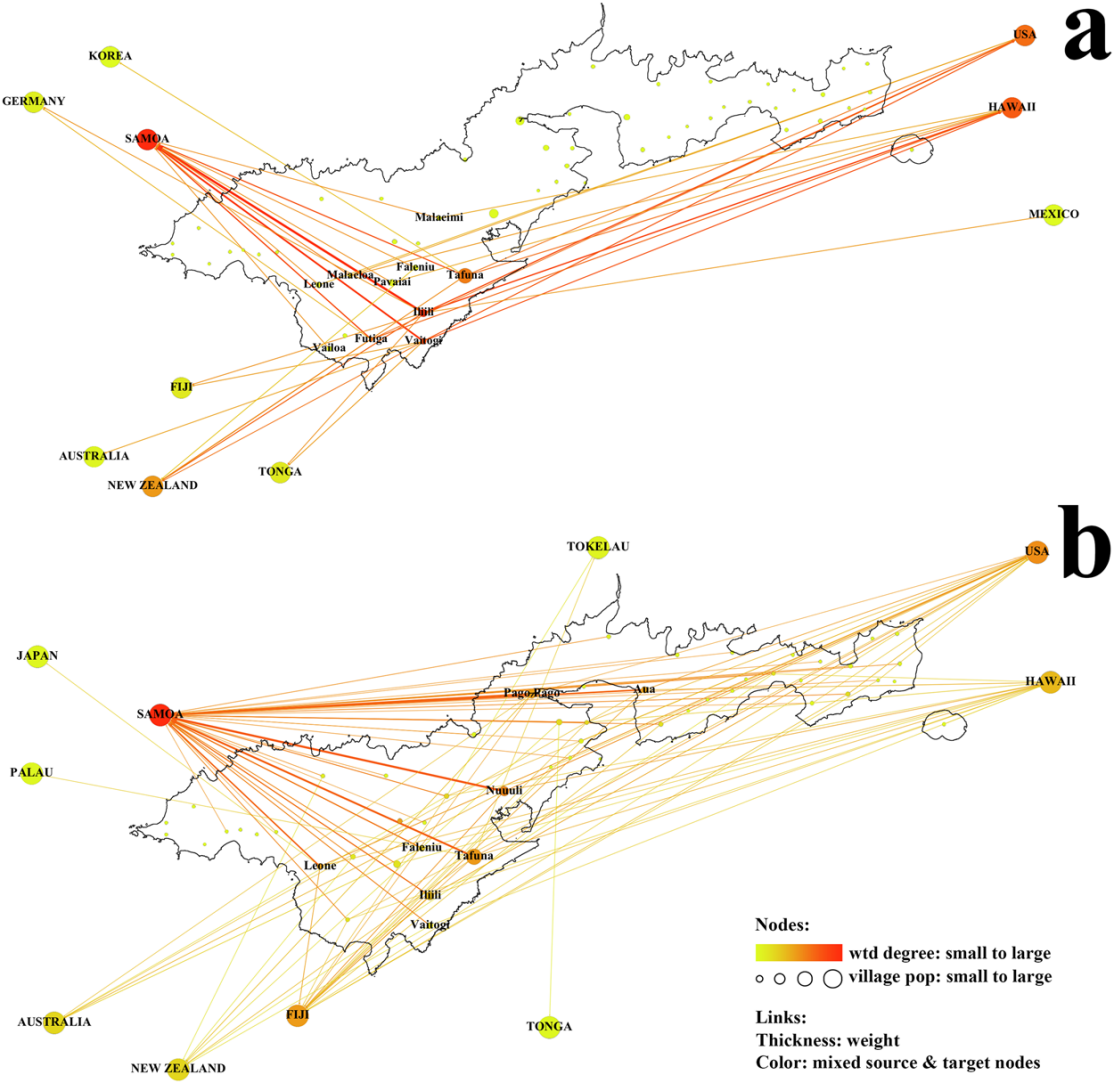
Network B - Overseas travel, 2014: (**a**) 8–13-year-old school children (travel in their lifetime); (**b**) adult workers (travel in the past 12 months). The size of dots for overseas countries does not reflect the country’s population, and location of dots are not to scale. The colour of lines is an average of weight of source and target nodes. wtd degree: weighted degree.

In 2014, children aged 8 to 13 years at an elementary school were asked whether they had ever (in their lifetime) travelled to Samoa or other overseas destinations, and how many times to each. An overseas travelling network for school children is shown in Fig. 3(a). For school children, the overseas travelling ratio was 57.0% (192/337) for all children, 59.9% (91/152) for males and 54.6% (101/185) for females (no significant difference between males and females on Poisson test).

#### Adult workers

In 2014, 245 respondents of 670 adult workers reported a total of 362 overseas trips in the past 12 months, indicating an overseas travelling ratio of 36.6% per year. The travelling network from the residence villages to the overseas countries was produced in Fig. 3(b), with a degree centrality analysis in Supplementary Fig. S7. Those who travelled overseas resided in 41 villages in American Samoa, with the most important hubs in Nuuuli, Tafuna, MapusagaFou and Pago Pago. Besides one unspecified-gender respondent, there were 88 male and 156 female travellers. The annual travelling ratio was 35.5% (88/248) for males and 39.2% (156/398) for females. There was no significant difference in the overseas travelling ratio between male and female adult workers. The age structure of the overseas travellers was analyzed and compared to the general age structure of the 2010 census population (Supplementary Fig. S8(a)). About 64.5% of those who travelled overseas were aged 35–54 years, which could be a reflection of the large number of cannery workers in this age group rather than a higher travelling ratio in this group. To test this theory, the travelling ratios by age group were analyzed in Supplementary Fig. S8(b), and no significant difference was found between age groups.

**Figure 4:**
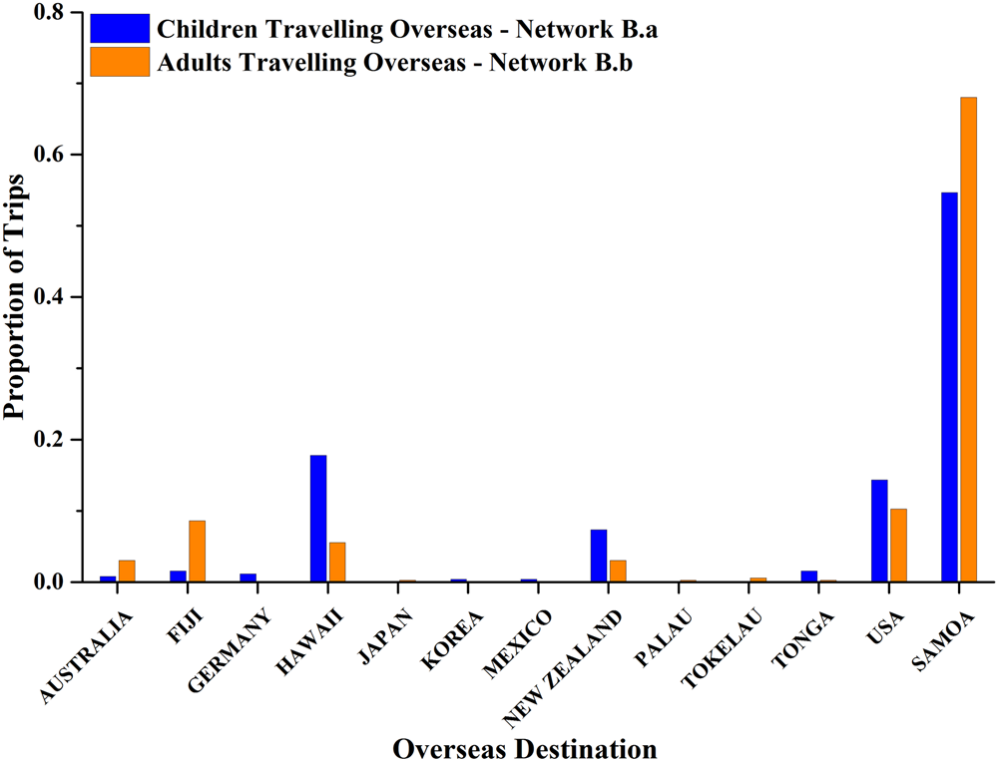
Overseas travel destinations 2014 - children (blue) and adults (orange). Network B.a is the overseas travelling of children (see Fig. 3(a)) and network B.b is the overseas travelling of adult workers (see Fig. 3(b)).

The destinations of the overseas travellers (school children and adult workers, Fig. 4) were analyzed. For both groups, Samoa dominated the travelling destinations (54.7% for school children, 68.1% for adult workers). Samoa (compared to other countries) was proportionally a more frequent destination for adults than children (*RR* = 1.25 and *p* = 0.04). Compared to children, adult workers also reported a higher proportion of trips to Fiji (*RR* = 5.55 and *p* < 0.001), but a smaller proportion to Hawaii (*RR* = 0.31 and *p* < 0.001) and New Zealand (*RR* = 0.41 and *p* = 0.02).

**Figure 5:**
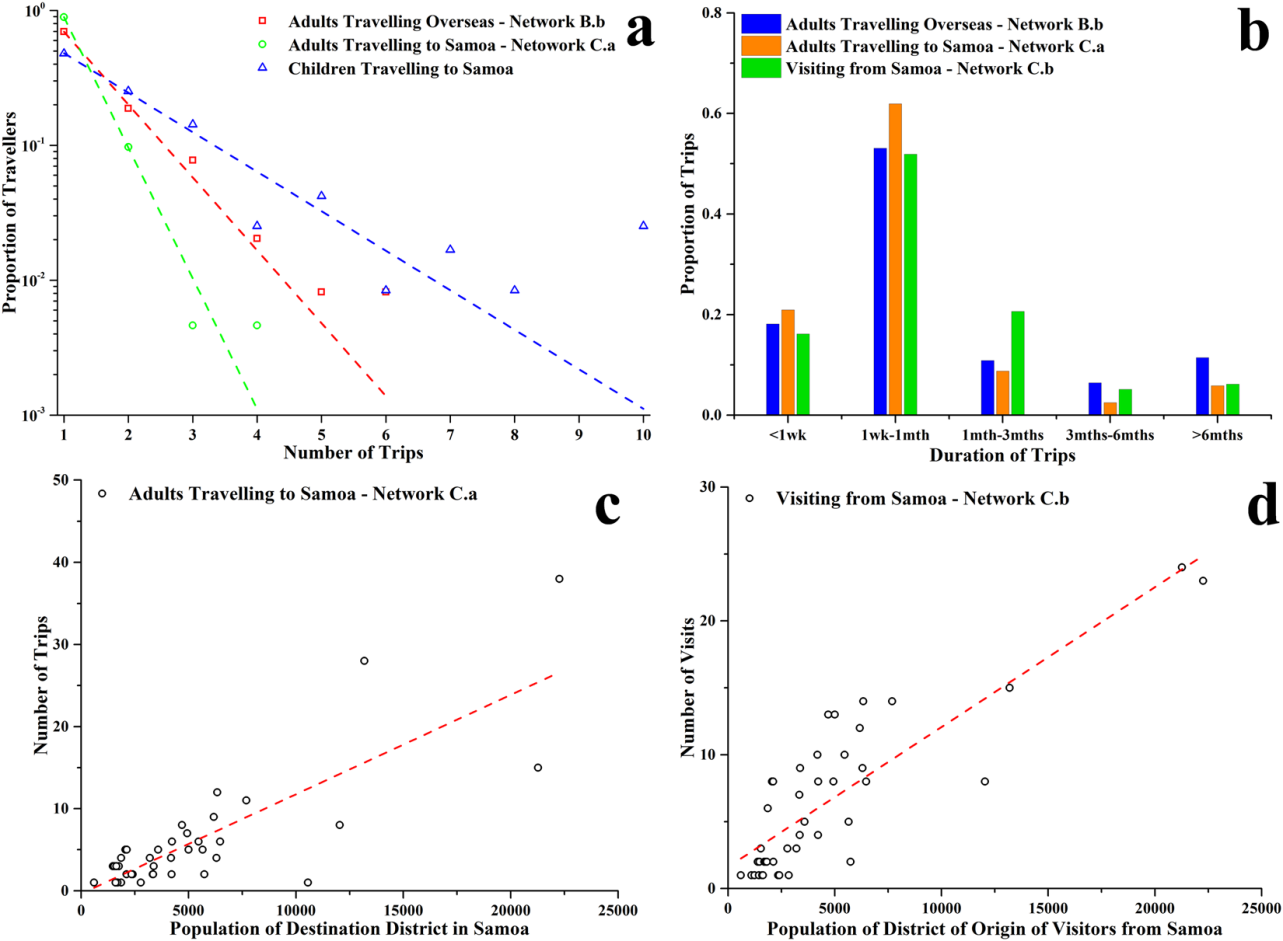
Travelling duration and frequency, 2014: (**a**) frequency of overseas travel (all countries and Samoa): number of trips per year for adults, and number of trips over lifetime for children; the dashed lines are the exponential fits; (**b**) duration of overseas trips (all countries and Samoa) and duration of stay of visitors from Samoa; (**c**) correlation between the number of trips from American Samoa to specific locations in Samoa and the destination district population; and (**d**) correlation between the number of visits from Samoa to American Samoa and the visitors’ residence district population in Samoa. Network C.a is travelling to Samoa (see Fig. 6(a)) and network C.b is visiting from Samoa (see Fig. 6(b)).

The travelling frequency (number of overseas trips) of the adult workers varied from one to six in the past 12 months (Fig. 5(a)), with no significant difference between males and females. The frequency indicated a good fit to the exponential function *y* = *a* × *e*^−*bx*^ (*a* = 2.415, *b* = 1.244, *R*^2^ = 0.998). No correlations were found between the travelling frequency and the traveller’s age for either the adult workers or school children.

The travelling duration and frequency of adult workers are shown in Fig. 5(b); 71.2% of trips were less than one month, with most people (53.1%) travelling for between one week and one month. The overseas travelling ratios by residence village were analyzed in Supplementary Fig. S9, and no significant difference was found between villages in the overseas travelling ratio using the Poisson test.

The travelling frequency of adult workers to Samoa in the last twelve months was found to follow an exponential distribution *y* = 8.307 × *e*^−2.230*x*^ (*R*^2^ = 0.9999, Fig. 5(a)). For school children, the number of trips to Samoa during their lifetime also followed an exponential distribution *y* = 0.947 × *e*^−0.675*x*^ (*R*^2^ = 0.985, Fig. 5(a)).

### Network C - Connectivity between American Samoa and Samoa

**Figure 6:**
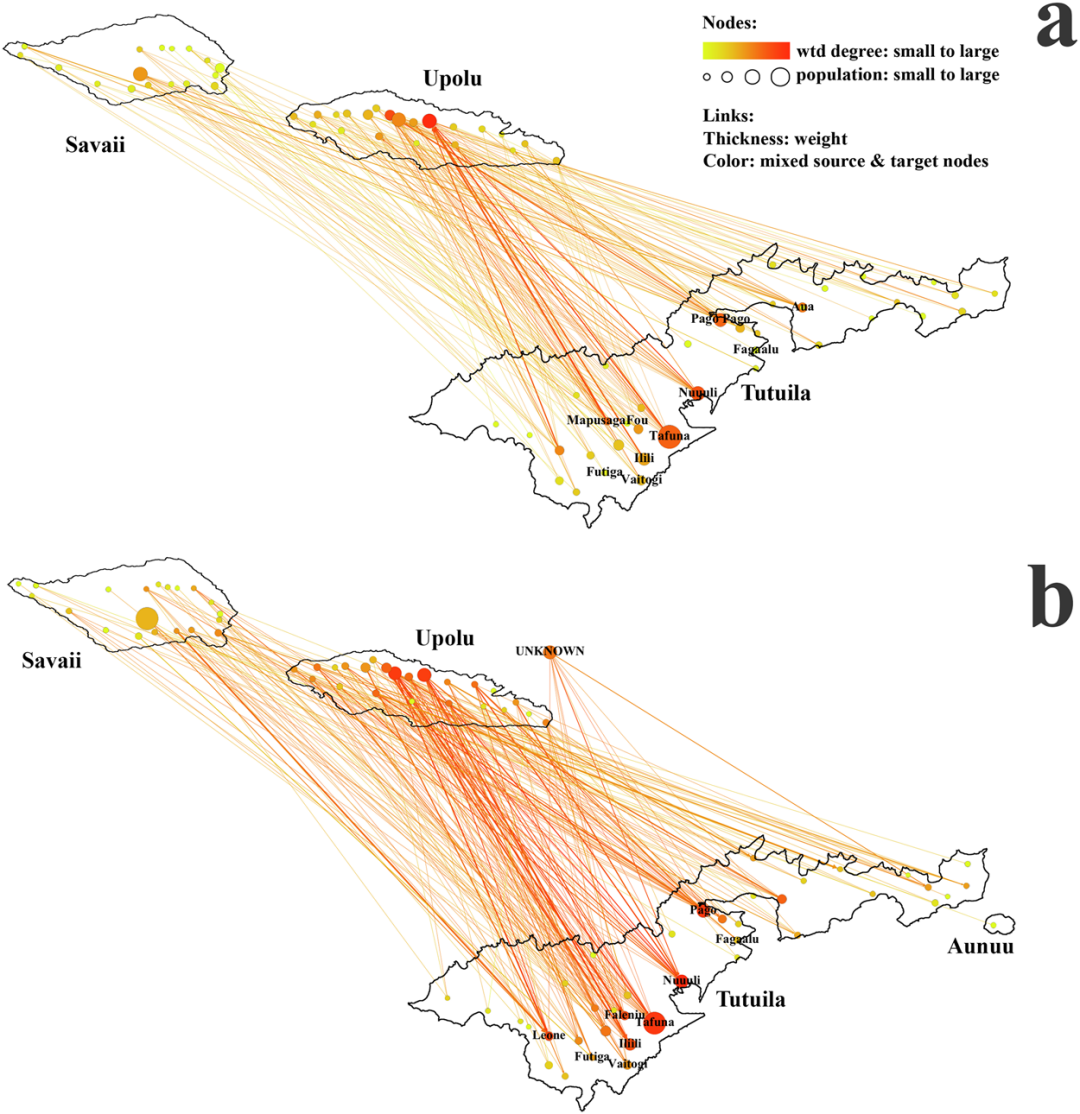
Network C – Connectivity between American Samoa and Samoa, 2014: (**a**) American Samoa residents (adult workers) travelling to specific locations in Samoa; (**b**) short-term visitors from specific locations in Samoa staying at homes of American Samoa residents. Note: maps of Samoa and American Samoa are not shown to scale. The colour of lines is an average of weight of source and target nodes. wtd degree: weighted degree.

Samoa dominated the overseas travel destinations of people living in American Samoa. In 2014, 218 (32.5%) of the surveyed 670 adult workers from the employment clinic and the cannery reported 245 trips to specific locations in Samoa, of which 242 trips were able to be accurately located on a country map. Destinations included 40 of the total 48 districts in Samoa, as shown in Fig. 6(a). The degree centrality^30^ is given in Supplementary Fig. S10(a). The large heterogeneity in the degree centrality indicated a large number of overseas travellers originating from a relatively small number of villages (Nuuuli, Tafuna, Pago Pago, MapusagaFou, etc). However, this was mainly affected by the population size variation. The linear regression analysis in Fig. 5(c) suggests a significant positive correlation (*y* = 1.21 × 10^−3^*x* − 0.346) between the number of trips from American Samoa to Samoa and the population of the destination district in Samoa, i.e. larger districts were more frequently visited. 82.8% of the trips were less than one month, with most people (61.9%) travelling for one week to one month (Fig. 5(b)).

Visits from Samoa to American Samoa were also investigated; the network is shown in Fig. 6(b) and the degree centrality analysis in Supplementary Fig. S10(b). Figure 5(d) shows a positive correlation (*y* = 1.05 × 10^−3^*x* + 1.615) between the number of visitors from a district in Samoa and the district’s population. 68.0% of the trips were less than one month, with most people (51.9%) travelling for one week to one month (Fig. 5(b)).

## Discussion

This study is one of the first to use survey data to quantify the extent of population movement over different geographical areas and time scales in the Samoan Islands. Three types of population mobility in the Samoan Islands were investigated using survey data from adult village residents, adult workers and school children collected in 2010 and 2014. A comprehensive picture has been built from the perspective of American Samoa residents of the extent of travel within American Samoa, including daily movements (commuting) within the main islands of American Samoa (Tutuila and Aunu’u), and residential relocation between villages in American Samoa. We also investigated recent short-term travel to overseas countries (including Samoa), and the extent of short-term visits from Samoa to American Samoa.

The findings indicate that given the extent of daily commuting between villages and the high connectivity of worksites, the transmission of pathogens between people and villages within American Samoa is extremely likely, including the relatively isolated villages in both extreme ends of the main island. Approximately half of adults reported being in paid employment, with a few main employer locations in the commercial hubs of Pago Pago and Fagotogo, Atu’u (location of the tuna cannery) and Tafuna (commercial hub, airport, and many government and business offices). The daily commuting covered the whole of Tutuila island, with work hubs drawing workers from villages all over the island. On the other hand, permanent residential relocation between villages in American Samoa was very infrequent, with most relocations being movements to larger villages. The relatively important role of frequent short-term mobility such as daily commuting, in addition to larger scale less frequent mobility, in disease transmission is increasingly being recognized. Intense local mobility networks can quickly amplify the impact of less frequent but larger scale or inter-country disease importation or exportation events.

Both adult workers and children in American Samoa travel overseas frequently. Approximately one third of the adult population makes one or more visits of up to several weeks per year to neighbouring countries, especially Samoa with which there are strong family, cultural, and commercial ties. The family ties mean that visitors also come from many locations in Samoa to villages throughout American Samoa. Children also travel frequently to a wide range of overseas destinations, mainly Samoa, Hawaii and mainland USA. Although females and older adults appear more likely to work in the cannery than males, there was no evidence that the frequency of trips or their duration varied by age or gender.

This study did not identify any specific locations in either American Samoa or Samoa with unexpectedly high frequency of travel between them. The overseas travelling ratio from villages in American Samoa was dependent on the village size, suggesting that all villages are similarly connected with overseas countries. Similarly, the numbers of trips by travelling destination (district) in Samoa were significantly positively correlated with the population in the district. We did not identify any villages or destinations which were more intensely connected than others, and would lend themselves to targeted surveillance or intervention, with the possible exception of transit points such air and sea ports. These findings will be useful for future estimates and projections of human movements between American Samoa and Samoa used in infectious disease modeling.

The travel patterns identified in this study have important implications for the transmission and spread of infectious diseases the Samoan islands. Our main motivation for developing this social network model was to improve understanding of the impact of population mobility on the elimination of lymphatic filariasis (LF). LF is a chronic infectious disease that has a years-long pre-patent infectious period when people may be unknowingly transmitting infections to others in countries with the appropriate vectors. As discussed by Ramaiah^31^, it is important to consider the impact of population movement on LF elimination because people may become infected during visits to endemic countries, be exposed to infected visitors from endemic countries, or miss opportunities for preventive interventions through absence from home during mass drug administration programs. Undocumented, temporary or seasonal migrants may not have access to the programs, or be absent when distribution occurs^32^. In American Samoa, relocations between villages are less likely to be an important factor as they are quite infrequent events. Since the main mosquito vectors for LF in the Samoan islands are predominantly day-biting and have a short flight range (100 meters^33,34^), daytime movements and daytime locations of people are important determinants of the transmission patterns and geographic distribution of LF in the long-term. In American Samoa, the high intensity of daily commuting across the whole of the main island will facilitate the geographic spread of infectious diseases. In the context of LF elimination, this could enable any residual foci of infection to spread island-wide over time.

Visits to non-endemic countries such as Hawaii and mainland USA do not increase the risk of LF transmission in American Samoa, but could reduce opportunities to participate in mass drug administration programs. Visits to other LF endemic countries, predominantly Samoa, would increase the likelihood that LF parasites are regularly exchanged between the two Samoas, resulting in a common pool of infection in the worm population that would make it more difficult to eliminate LF without cross-border collaborative action. LF elimination programs need to consider social connectivity between countries, and implement cross-border strategies to reduce the risk of transfer and reintroduction of parasites, particularly if there is a large discrepancy in infection prevalence between highly connected places. At present, it is not known whether the infection prevalence in the general population is higher in Samoa or American Samoa, so we cannot say in which direction infections are likely to flow.

There are a number of limitations of the study that need to be considered. Firstly, we used an egocentric network (wherein a subject is asked to identify his or her social contacts and their relationships) and there may be connections for which we have no data. Second, recall bias regarding travel history may be an issue. Third, the data were collected from cross-sectional surveys and movements may change over time. Fourth, the study focuses on movements of defined target populations and may not be representative of the population at large.

The current study shows that populations in the remote Samoan islands are highly mobile, and has quantified the extent of their mobility as well as the destinations. The extensive movement of people between the Pacific islands is believed to have contributed to recent rapid spread of Zika^35^ and Chikungunya viruses, and the repeated epidemics of dengue across the region.

The social networks developed in this study could be used to investigate the role of human mobility within and between the Samoan Islands over different time scales, which are important in the transmission of infectious diseases, including those with long incubation and infectious periods. Social network analysis^30^ (SNA) has so far mostly been used to model infectious diseases that are transmitted through respiratory secretions (e.g. influenza^36^), faecal-oral route (e.g. typhoid^37^) or sexual contact. SNA has also been used to investigate the role of movement between households in the transmission of dengue (a vector-borne disease with short incubation period and duration of infectivity), which is prone to explosive but relatively short-lived epidemics^38^. However, to our knowledge, high-resolution SNA has not been used to model the combined effects of both short and long term mobility, and connectivity at different spatial scales (between villages, districts and countries) on infectious diseases with long incubation periods, long duration of infectiousness, and relatively low infectivity, such as LF. Social networks might also be important for implementation of disease control programs, and SNA has been used to investigate the role of social networks in the acceptance of mass drug administration, the major public health intervention for LF^39^. Using the frameworks and findings from this study, future models will be specifically designed to understand the potential influence of population mobility on transmission of LF within and between the Samoan islands, and provide information to improve the progress towards elimination of this disease.

## Materials and Methods

### Study location

American Samoa is an unincorporated US Territory in the south Pacific, with a population of 55,519 in the 2010 population census^40^. The Samoan islands were colonized in 1899, when the Tripartite convention^41^ divided the Samoan islands into a German colony known at the time as Western Samoa, and the US territory of American Samoa. From 1914, Samoa was a Trust Territory of New Zealand until independence in 1962.

People born in American Samoa are American nationals, but are not US citizens unless one of their parents is a US citizen. US nationals have the right to reside in the USA and may apply for naturalization after three months of residency. American Samoa’s airport in the capital, Pago Pago, can be reached by flight only from Hawaii (twice weekly) or Apia, Samoa (numerous daily flights). Ferries also travel between Pago Pago and Apia weekly. Citizens of Samoa need a visa to enter American Samoa for work, education or visiting friends and family; immigration requires a local sponsor. In the other direction, tourist visas are not required for American Samoans for visits of up to 60 days to Samoa. Samoa has numerous flight connections to New Zealand, Fiji, and Australia as well as Pago Pago, American Samoa.

### Data Sources and Analysis

Data on population movements were obtained from two survey datasets from American Samoa, a cross-sectional seroprevalence study in 2010^42^ and the follow-up test and treat study in 2014^23^. Both studies investigated the prevalence of LF antigens and antibodies following mass drug administration, and study design and findings have been described in previous publications^11,23^. Briefly, the 2010 study was a community-based representative sero-survey of all adults using a serum bank collected on the main island of Tutuila (where over 95% of the population live), the adjacent island of Aunu’u, and the remote Manu’a islands. In 2014, a second survey was conducted in American Samoa to confirm the presence of putative geographical ‘hotspots’ and high-risk population groups for LF transmission, such as recent migrants from Samoa. Field data were collected from: (1) adult workers (aged ≥15 years), recruited from a Department of Health clinic where workers attend for fitness-for-work medical examinations and from the Starkist tuna cannery (the largest private employer in the territory); (2) school children (Grades 3 to 8, aged 8 to 13 years), from the large elementary school in one suspected hotspot area, to obtain a larger sample of children living nearby.

During these two surveys, respondents provided information about their work locations (2010 and 2014) and their movements to and from Samoa and other countries (2014 only). The 2010 survey collected data on demographics, and household and workplace locations of 807 adults in a geographically representative sample of 659 households. Ages of participants ranged from 17 to 87 years of age (mean 40 years), 52% were males, 97% were of Samoan ethnicity, and 96% had lived in their current home for more than 3 years. Data on the villages of residence and villages of workplaces were used to build the commuting network within American Samoa.

From the 2014 survey, data from 1,007 participants were used in this study, comprising 670 adult workers aged 16 to 68 years (172 employment clinic attendees and 498 tuna cannery workers), and 337 school children. A detailed description of the design of the survey can be found in Lau *et al*.^23^. Tuna processing is American Samoa’s primary industry, and sampling was conducted in the cannery as a convenient method of reaching a large sample of adults who lived all over the island of Tutuila. In 2010, 8.2% of the total employed persons were working in the tuna cannery, and the ratio varied from 8.2% to 28.6% in recent years^40^, with an average of 17.7%. In 2014, data were again collected on demographics, and household and workplace locations. In addition, data on travel history were also collected. Adult workers were asked if they had been to Samoa (data on the village of destination and the travelling duration were collected) or received any visitors from Samoa (data on the residence village of the visitors and the visiting duration were collected), and if they had been to other countries in the last twelve months (data on the country of destination and the travelling duration were collected). School children were asked if they had ever been to Samoa and other countries (data on the country/countries of destination was collected). These data were used to build the travelling/visiting network between American Samoa, Samoa and other countries, as well as the daily commuting network within American Samoa.

The durations of trips were recorded in 5 categories (<1 week, 1 week to 1 month, 1 to 3 months, 3 to 6 months, and >6 months.) Duration of trips was estimated by taking the mid value of each of these intervals as an estimate of length of each trip.

Data entry was done in Qualtrics^43^. Data management, cleaning and initial descriptive analysis were done in in STATA 14 and Microsoft Excel.

### Mobility Networks and Visualization

Three types of mobility networks were investigated, in terms of the nature of population movements (Table 1):

- Movement within American Samoa (Network A). Daily commuting between home and work within American Samoa, and relocation of village of residence within American Samoa (i.e. moving from one village to another).
- Movement from American Samoa to other countries (data from children aged 8–13 years and adult workers, 2014; Network B).
- Movement between specific locations in American Samoa and Samoa (Network C). Travel between American Samoa (villages) and Samoa (districts), including trips made by residents of American Samoa to Samoa, and short-term visitors from Samoa to American Samoa.

**Table 1:** Summary of social networks.

Network Label	Data Source	Population	Nodes	Links
Network A – Daily commuting within American Samoa. Figure 1.	2010	Adult workers	Villages in American Samoa	Commuting (residence village to workplace village)
Figure S3. Daily commuting, cannery workers	2014	Cannery workers	Villages in American Samoa	Commuting (residence village to workplace village)
Figure S6. Relocation within American Samoa.	2014	Adult workers	Villages in American Samoa	Relocation (previous village to current village)
Network B(a) – Overseas travel, children. Figure 3(a).	2014	School children	Villages in American Samoa; Overseas countries	Travelling (American Samoa to overseas countries)
Network B(b) – Overseas travel, adults. Figure 3(b).	2014	Adult workers	Villages in American Samoa; Overseas countries	Travelling (American Samoa to overseas countries)
Network C(a) – Travel to Samoa. Figure 6(a).	2014	Adult workers	Villages in American Samoa; districts in Samoa	Travelling (American Samoa to Samoa)
Network C(b) – Visitors from Samoa. Figure 6(b).	2014	Adult workers	Villages in American Samoa; districts in Samoa	Visiting (Samoa to American Samoa)

The mobility networks were formulated by creating the weighted links from source nodes (place of origin) to target nodes (place of destination), where each node was an administrative division (village, district, or country). In American Samoa, all reported nodes for residence or work were villages (or combined groups of 2 to 3 small villages). The questionnaires also requested names of destination or visitor source villages in Samoa, which were provided in most cases, but the (American Samoan) respondents occasionally reported district names or an island (Savaii) instead of a specific village name in Samoa. The Samoan villages were aggregated by districts (or the island of Savaii) in the analysis as the sample size was not sufficient for village-level analysis.

The weights for each link were proportionate to the reported number of population movement events between the nodes. The approach produced a directed weighted network^30^ *G* = (*V*, *E*), where *V* is the node set and *E* is the edge set. For any directed link from node *i* to node *j*, the link is denoted *l*_*ij*_ = (*i*, *j*) and the weight of the link is denoted *w*_*ij*_. The node *i* is called the precursor of node *j*, and correspondingly the node *j* is called the successor of node *i*. For any node *i* ∈ *V*, the out-degree and in-degree^30^ of node *i* are respectively denoted ${\deg }^{+}(i)=|{N}^{+}(i)|$ and ${\deg }^{-}(i)=|{N}^{-}(i)|$, where *N*^+^ (*i*) and *N*^−^(*i*) are respectively the precursor set and the successor set of node *i* and |*N*| is the cardinality of set *N*. The weighted (wtd) out-degree of node *i* is denoted ${{\rm{wdeg}}}^{+}(i)={\sum }_{j\in {N}^{+}(i)}{w}_{ij}$ and the weighted in-degree of node *i* is denoted ${{\rm{wdeg}}}^{-}(i)={\sum }_{j\in {N}^{-}(i)}{w}_{ji}$. The degree of node *i* is $\deg (i)={\deg }^{+}(i)+{\deg }^{-}(i)$ and the weighted degree is wdeg(*i*) = wdeg^+^ (*i*) + wdeg^−^(*i*).

Each node in the networks was assigned several attributes to denote the uniqueness (Id and Label), geographic location (Lat. and Long.), and the census population (Pop). For the villages in American Samoa, the geographic locations were available from the land use data from the Department of Commerce^44^ and the latest census populations were obtained from the 2015 Statistical Yearbook^40^. For Samoa, geographic information systems (GIS) and population census data were obtained from the Samoa Bureau of Statistics^45^, and the geographic locations of districts were approximated by using the centroids of the district polygons.

The networks were visualized with Gephi, an open source software for graph and network visualization^46^. Gephi also enables basic analysis of the networks, e.g. degree distribution, modularity^47^ and clustering coefficient^48^. In this study, mobility networks were visualized with the geographical layout, e.g., most nodes in the networks were arranged geographically and overlaid on maps of American Samoa and Samoa.

### Data Availability

Some access restrictions apply to the data underlying the findings. American Samoa has a very small population, and high resolution geo-referenced data would potentially allow identification of individuals and households, and breach confidentiality. The datasets generated during and/or analysed during the current study may be available from the corresponding author on reasonable request providing confidentiality restrictions are met.

### Ethics Statement

The 2010 study received ethical clearance from the American Samoa Institutional Review Board, the Medical Research Ethics Committee of The University of Queensland (2010000114), and Queensland Health Forensic and Scientific Services Human Ethics Committee (HREC/10/QFSS/1). Permission was also sought from the Department of Samoan Affairs and village chiefs before village visits. Ethics approvals in 2014 were granted by the American Samoa Institutional Review Board, the Human Research Ethics Committees at James Cook University (H5519) and The University of Queensland (2014000409). The study was conducted in collaboration with the American Samoa Department of Health, and official permission for village visits was sought from the Department of Samoan Affairs and village chiefs and/or mayors. Verbal and written information on the study were provided in Samoan and/or English according to the participants’ preference, and written informed consent was obtained from all participants or their parent or guardian if under 18 years of age. All data were de-identified prior to analyses. All methods were performed in accordance with the relevant guidelines and regulations.

### Significant Statement

Infectious disease control and elimination programs need to consider social connectivity between countries, and implement cross-border strategies to reduce the risk of transmission of pathogens between countries. This research investigated population mobility in the Samoan Islands using survey data. The analysis indicated frequent and widespread movement of workers between villages within American Samoa through daily commuting between villages up to both extreme ends of the main island and the few worksite hubs. In addition, high connectivity between American Samoa and Samoa, and long trip durations, have significant implications for transmission of infectious diseases between the two places and for disease control programs. Data generated in this study will provide parameters for modeling disease transmission based on rates of population movement, which will affect the effectiveness of national programs designed to interrupt transmission, and the likelihood of elimination.

## Electronic supplementary material

Supplementary Information
