# Supplementary material for: The extensive networks of frequent population mobility in the Samoan Islands and their implications for infectious disease transmission
Source: Sci Rep. 2018 Jun 28;8:10136. doi: 10.1038/s41598-018-28081-x (PMC6031642; doi:10.1038/s41598-018-28081-x)
Supplement: Supplementary file 1 — Supplementary Information [file 41598_2018_28081_MOESM1_ESM.docx]

**The extensive networks of frequent population mobility in the Samoan Islands and their implications for infectious disease transmission**

**Author Affiliation:**

Zhijing Xu^a^, Colleen Lau^a,b^, Xiaoyan Zhou^c^, Saipale Fuimaono^d^, Ricardo J. Soares Magalhães^b,c^ , Patricia M Graves ^e^.

^a^ Research School of Population Health, The Australian National University, ACT 2601, Australia

^b^ Children’s Health and Environment Program, Child Health Research Centre, The University of Queensland, South Brisbane 4101 QLD, Australia

^c^ UQ Spatial Epidemiology Laboratory, School of Veterinary Science, The University of Queensland, Gatton 4343 QLD, Australia

^d^ Department of Health, Pago Pago, American Samoa

^e^ Australian Institute of Tropical Health and Medicine, College of Public Health, Medical and Veterinary Sciences, James Cook University, Cairns, Queensland, Australia

**Corresponding Author:**

Zhijing Xu

Building 62, Mills Road, The Australian National University, Acton, 2601

Tel: +61 02 6125 6803

Email: sting.xu@anu.edu.au

**Keywords:** population mobility, social network, disease transmission, lymphatic filariasis

1. **Mobility in American Samoa**


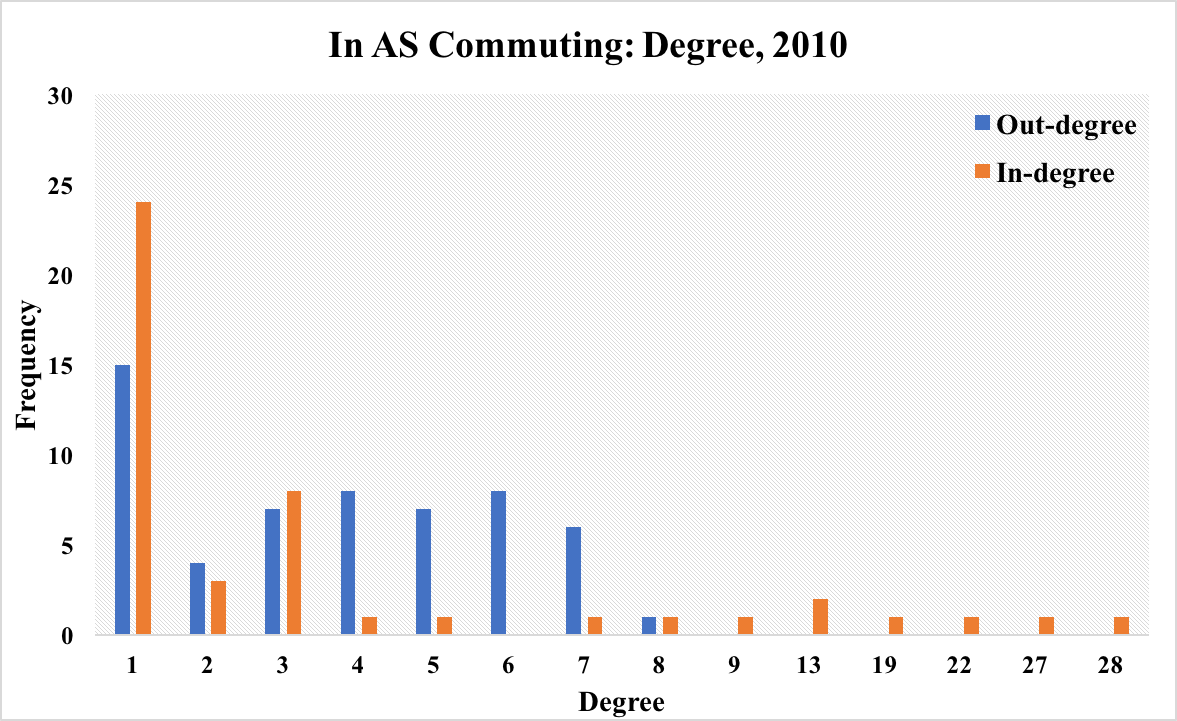

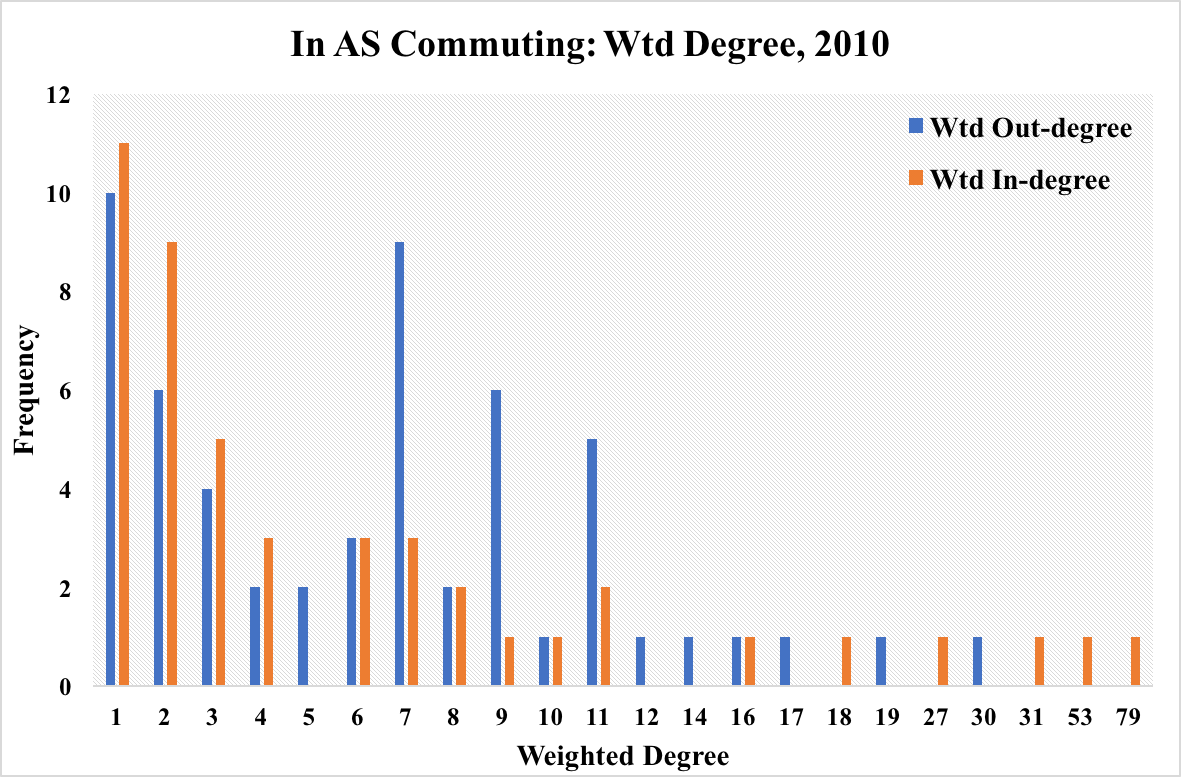


Figure S1. Degree centrality of villages in American Samoa, Network A, 2010







Figure S2. The age distribution of workers (a) and employment ratio by age group (b), 2010

Table S1. Significant differences in employment ratio between age groups

| Group X | Group Y | Rate Ratio | p value | 95% CI |
| --- | --- | --- | --- | --- |
| 15-24 | 25-34 | 0.44 | p < 0.001 | 0.30 - 0.63 |
| 15-24 | 35-44 | 0.39 | p < 0.001 | 0.27 - 0.54 |
| 15-24 | 45-54 | 0.43 | p < 0.001 | 0.30 - 0.61 |
| 15-24 | 55-64 | 0.53 | p = 0.002 | 0.36 - 0.80 |
| 25-34 | 65+ | 1.85 | p = 0.006 | 1.18 - 2.98 |
| 35-44 | 55-64 | 1.39 | p = 0.048 | 0.99 - 1.96 |
| 35-44 | 65+ | 2.12 | p < 0.001 | 1.37 - 3.37 |
| 45-54 | 65+ | 1.91 | p = 0.003 | 1.22 - 3.07 |

**
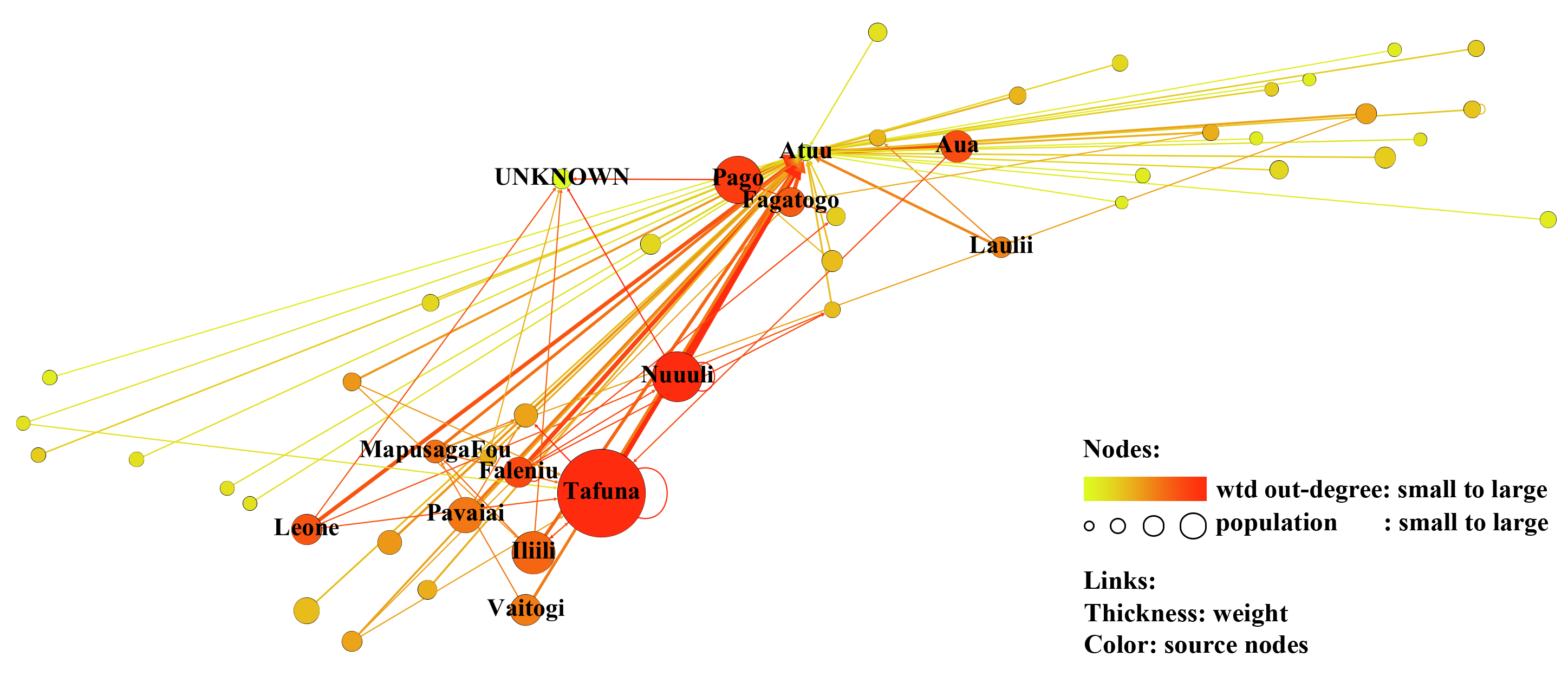
**

Figure S3. Commuting between residence and workplace village, cannery workers, 2014. The cannery is located in Atu’u village.

**
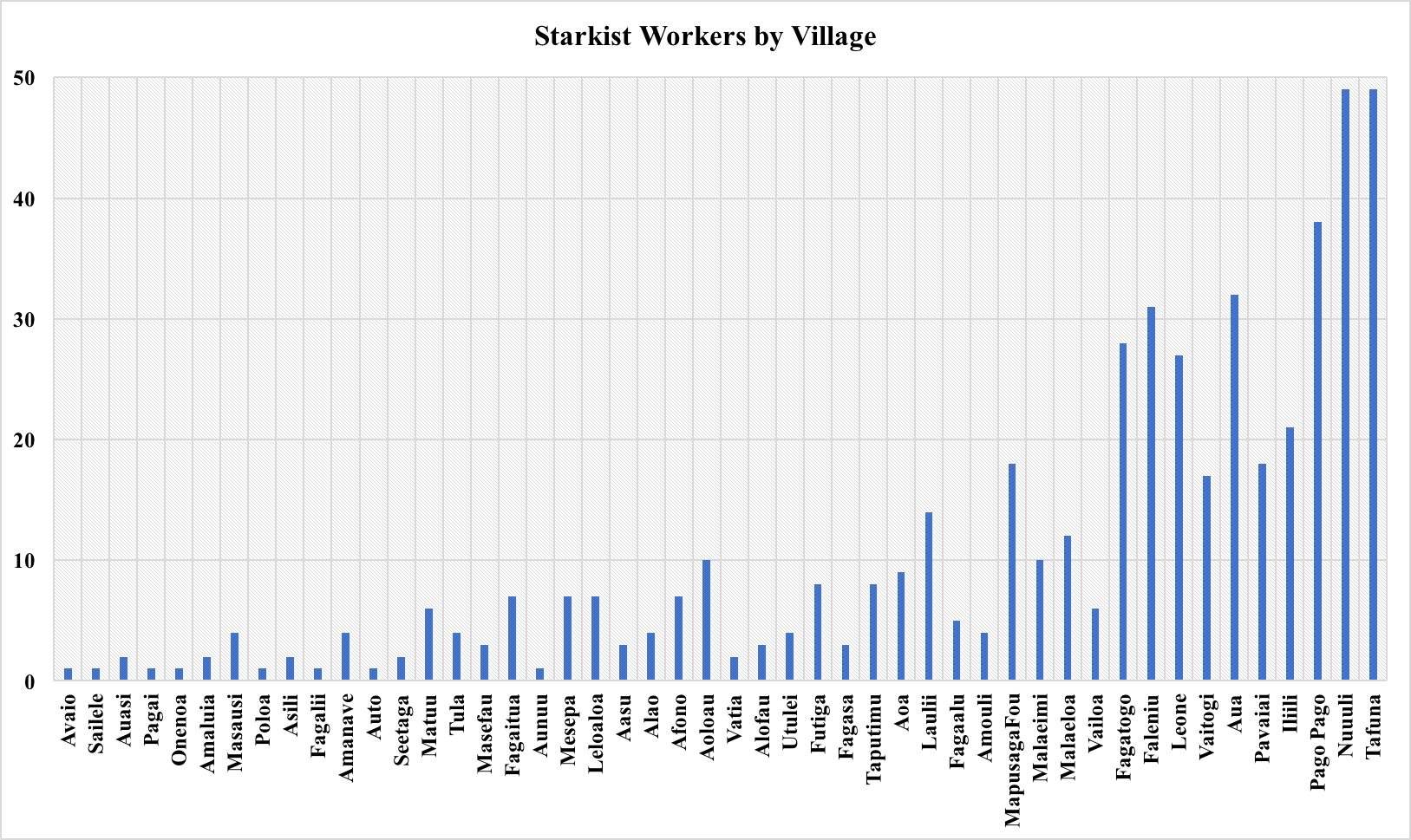
**

Figure S4. Cannery workers by village of residence, 2014

**



**

Figure S5. Cannery workers, 2014: (a) age structure, and (b) correlation between the number of cannery workers and the village population, $y=7.95\times10^{-3}x+1.898$.

**
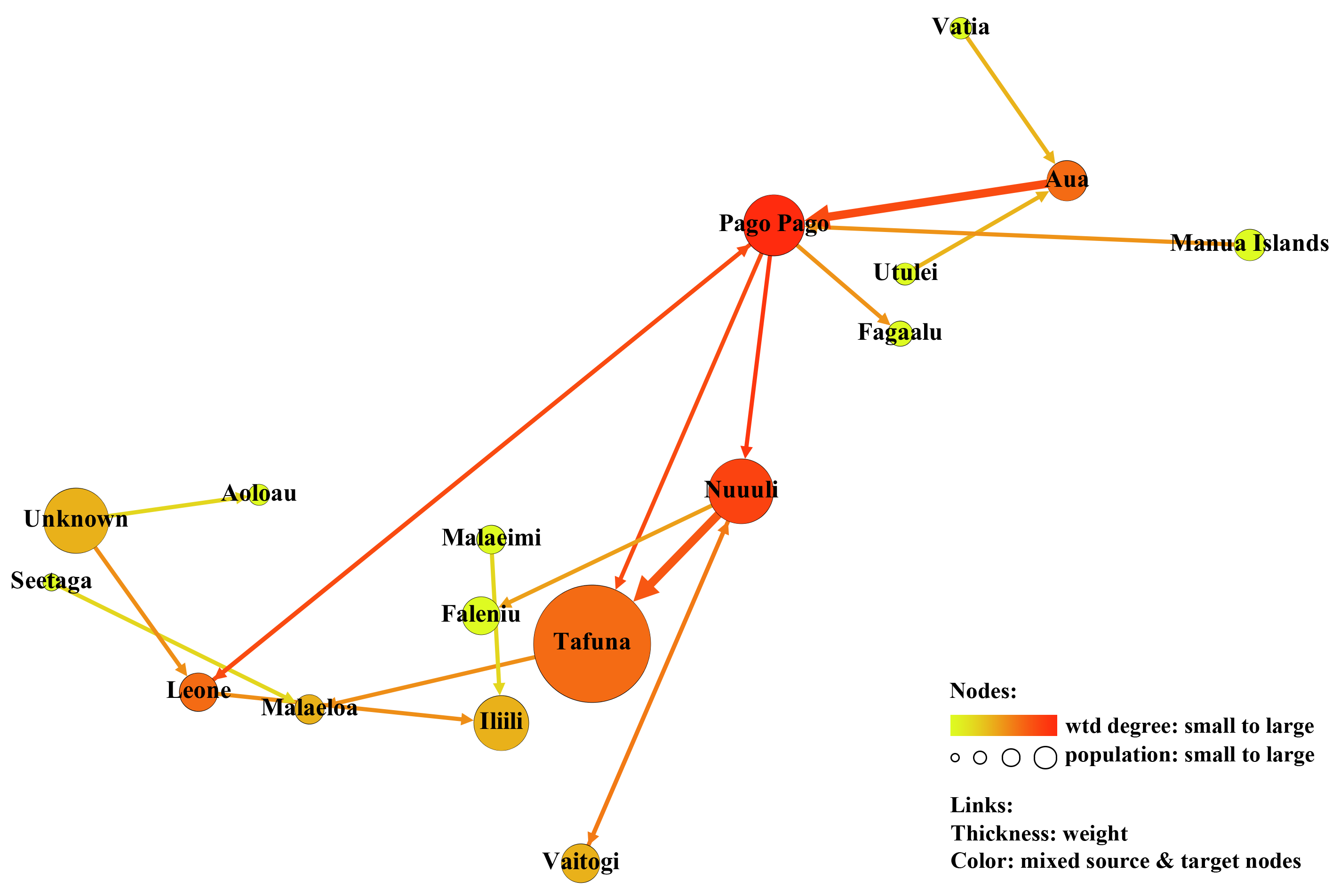
**

Figure S6. Residential relocation between villages in American Samoa, adult workers, 2014

1. **Traveling Overseas**


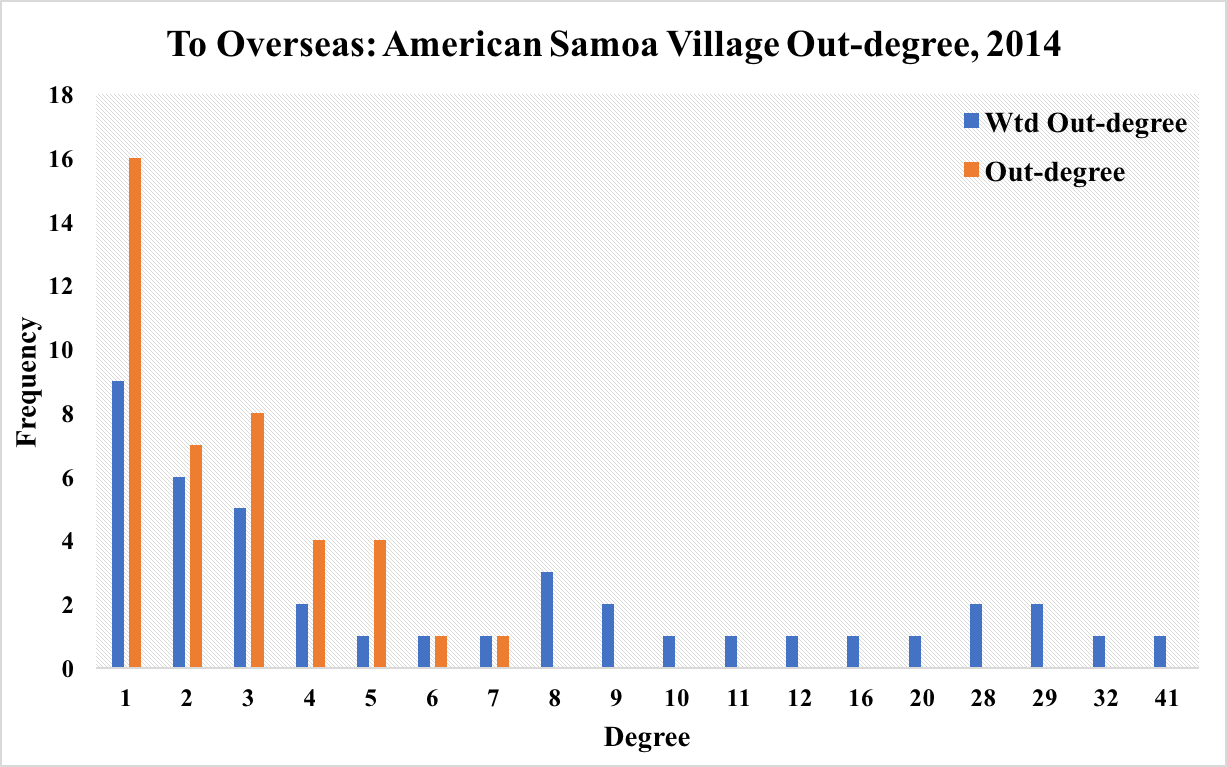


Figure S7. Degree centrality of villages in American Samoa, Network B.b

The travelling ratio was calculated by dividing the number of overseas travelers by the number of total respondents in each age group. There was no significant difference between the age groups in overseas traveling ratio.


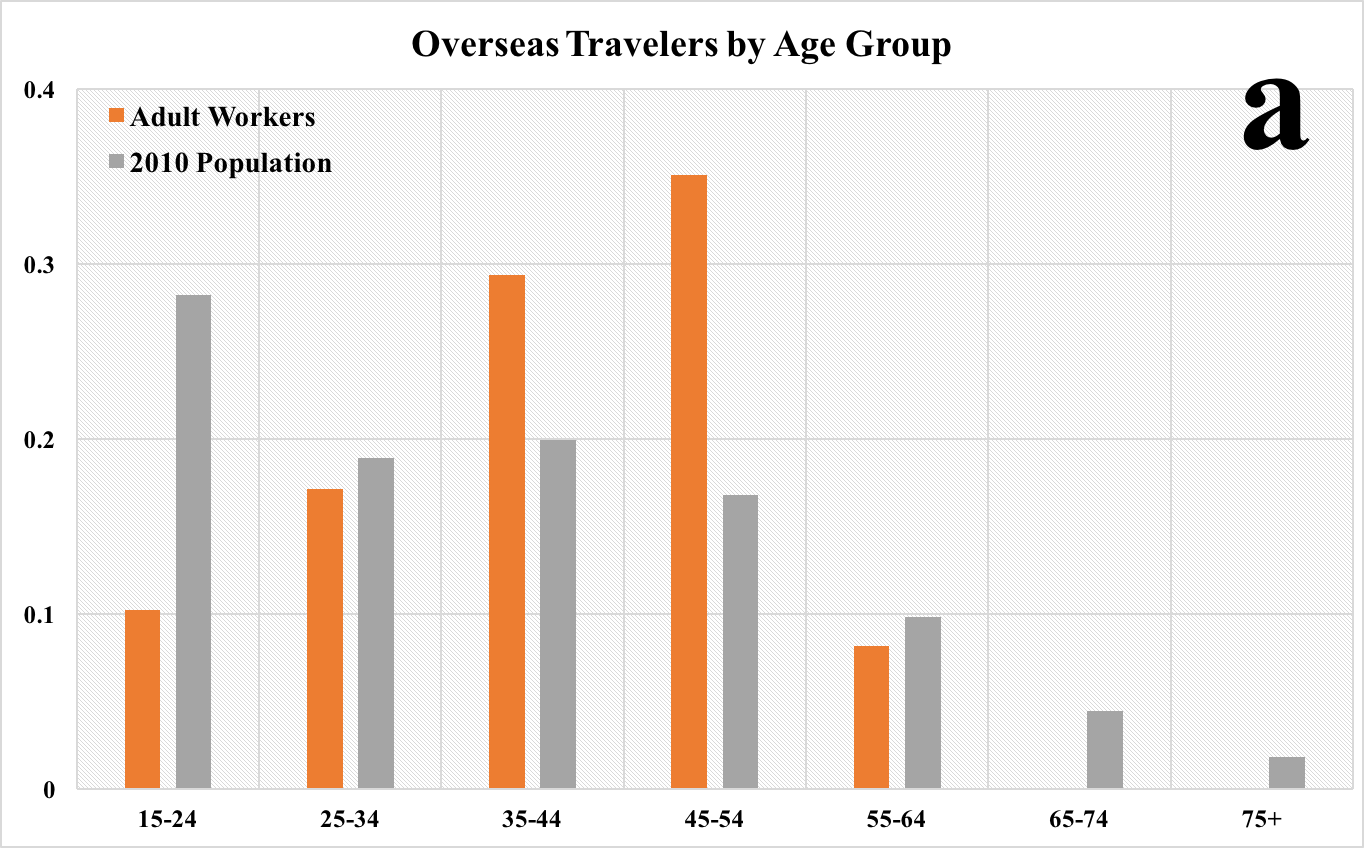

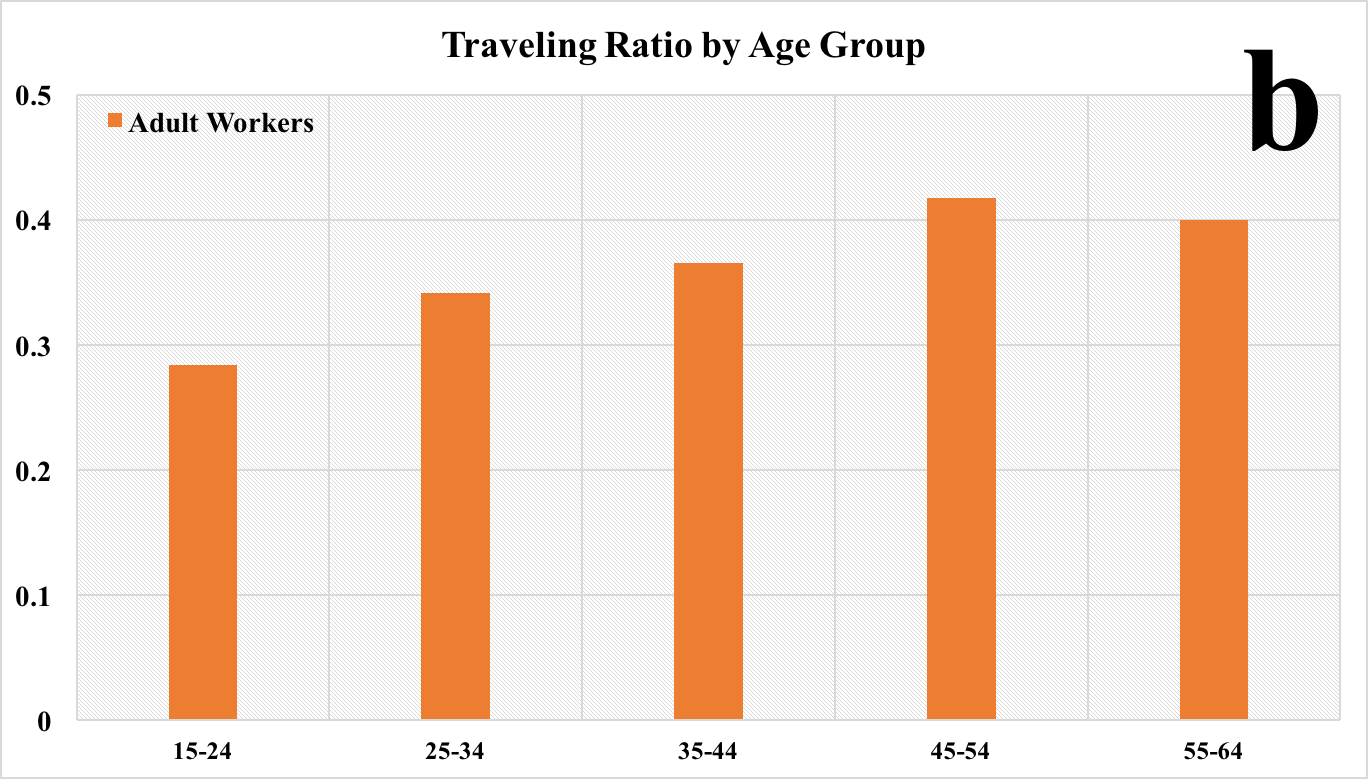


Figure S8. Age distribution of overseas travellers (a) and travelling ratios by age group (b), adult workers, 2014


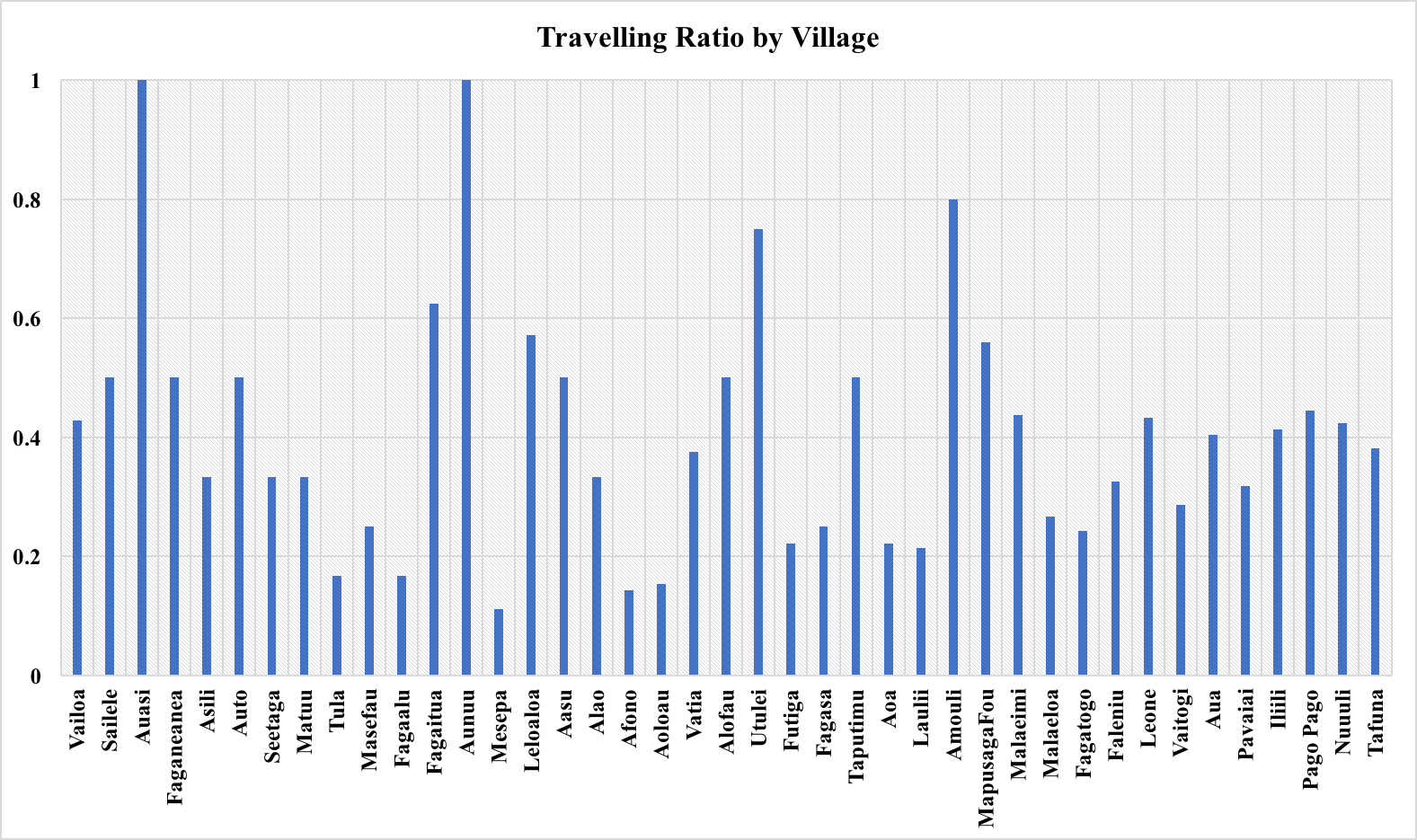


Figure S9. Overseas travelling ratio by village of residence, adult workers, 2014

1. **Travelling between American Samoa and Samoa**

**
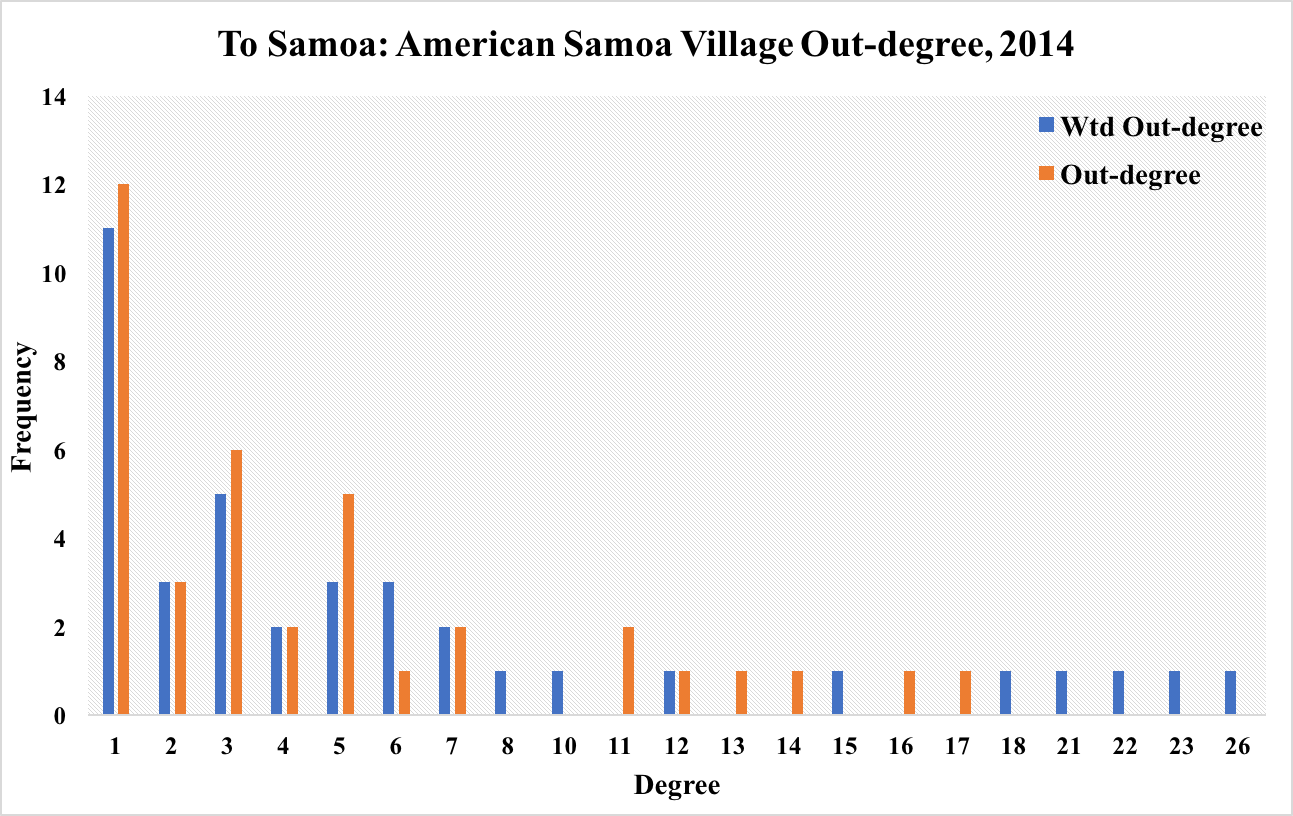

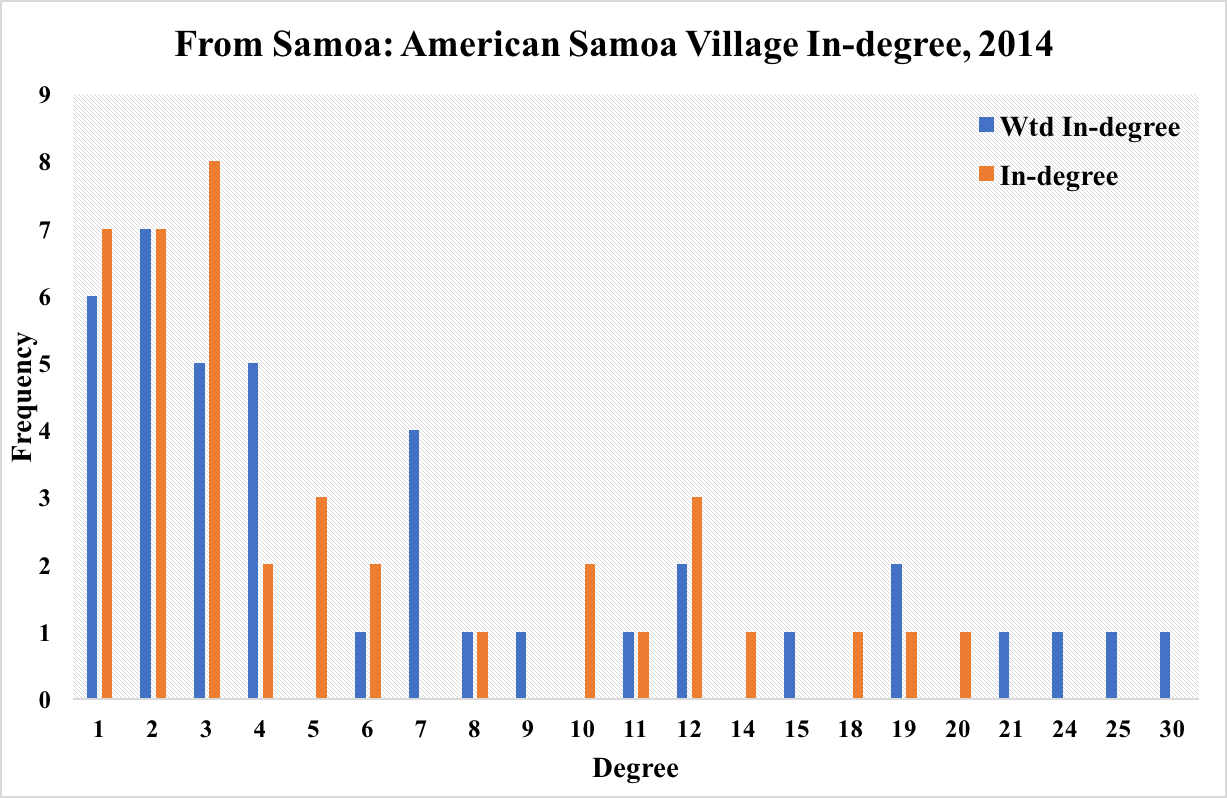
**

Figure S10. Degree centrality of villages in American Samoa, Network C. Out-degree: number of districts of Samoa connected to the village; Wtd Out-degree: number of trips from the village. In-degree: number of districts of Samoa connected to the village; Wtd In-degree: number of trips to the village.
